# Diabetes-Specific Complete Smoothie Formulas Improve Postprandial Glycemic Response in Obese Type 2 Diabetic Individuals: A Randomized Crossover Trial

**DOI:** 10.3390/nu16030395

**Published:** 2024-01-30

**Authors:** Pichanun Mongkolsucharitkul, Bonggochpass Pinsawas, Apinya Surawit, Tanyaporn Pongkunakorn, Thamonwan Manosan, Suphawan Ophakas, Sophida Suta, Sureeporn Pumeiam, Korapat Mayurasakorn

**Affiliations:** Siriraj Population Health and Nutrition Research Group, Department of Research Group and Research Network, Faculty of Medicine Siriraj Hospital, Mahidol University, Bangkok 10700, Thailand; pichanun.mon@mahidol.edu (P.M.); bonggochpass.pin@mahidol.edu (B.P.); apinya.sua@mahidol.edu (A.S.); tanyaporn.pon@mahidol.edu (T.P.); thamonwan.manosan@gmail.com (T.M.); phakhaun1234@gmail.com (S.O.); sophida.sut@mahidol.edu (S.S.); sureeporn.pum@mahidol.edu (S.P.)

**Keywords:** clinical nutrition, smoothie drink, meal replacement, medical food, diabetes mellitus, obesity

## Abstract

This study aimed to compare newly developed diabetes-specific complete smoothie formulas with a standard diabetes-specific nutritional formula (DSNF) regarding their effects on glucose homeostasis, insulin levels, and lipid metabolism in obese type 2 diabetes (T2DM) patients. We conducted a randomized, double-blind, crossover study with 41 obese T2DM participants to compare two developed diabetes-specific complete smoothie formulas, a soy-based regular smoothie (SM) and a smoothie with modified carbohydrate content (SMMC), with the standard DSNF, Glucerna. Glycemic and insulin responses were assessed after the participants randomly consumed 300 kilocalories of each formulation on three separate days with a 7-day gap between. Postprandial effects on glycemic control, insulin levels, and lipid metabolism were measured. SMMC resulted in a significantly lower glucose area under the curve (AUC_0–240_) compared to Glucerna and SM (*p* < 0.05 for both). Insulin AUC_0–240_ after SMMC was significantly lower than that after SM and Glucerna (*p* < 0.05). During the diets, the suppression of NEFA was more augmented on SM, resulting in a less total AUC_0–240_ of NEFA compared to the SMMC diet (*p* < 0.05). *C*-peptide AUC_0–240_ after SMMC was significantly lower than that after Glucerna (*p* < 0.001). Conversely, glucagon AUC_0–240_ after SMMC was significantly higher than that after SM and Glucerna (*p* < 0.05). These results highlight SMMC as the better insulin-sensitive formula, potentially achieved through increased insulin secretion or a direct reduction in glucose absorption. The unique composition of carbohydrates, amino acids, and fats from natural ingredients in the smoothies may contribute to these positive effects, making them promising functional foods for managing diabetes and obesity.

## 1. Introduction

The effective management of nutrition and food is crucial in achieving optimal glycemic control while providing adequate nutrients to meet metabolic demands and avoiding complications associated with type 2 diabetes (T2DM) and dysglycemia in people at risk for poor glucose control [1]. Hospitalized and ill individuals are especially vulnerable to infection and increased mortality due to hyperglycemia [2,3]. While diabetes nutrition guidelines advise how food choices can help achieve normal glycemic control, patients cannot always follow such diets due to illness and personal psychosocial and economic barriers [4]. Patients who are incapable of meeting their nutritional needs through oral dietary intake alone often require an enteral nutrition formula to be administered via a tube to facilitate nutrition goals.

Over recent decades, various enteral nutrition (EN) formulas have been developed for medical nutrition therapy [4,5]. “Standard” EN formulas mostly contain approximately 45–50% carbohydrate, 30–35% fat, and 15–20% protein of total energy intake and are widely accepted but can worsen hyperglycemia in diabetes patients [6]. In 2022, the American Diabetes Association (ADA) recommended low-carbohydrate (LC) or very-low-carbohydrate (VLC) eating plans for individuals with T2DM who do not meet glucose targets or require glucose-lowering drugs [7]. This aligns with a Nurses’ Health Study and Health Professionals Follow-up Study [8], which indicate that LC patterns, mainly with high-quality macronutrients, were significantly associated with lower mortality among adults with T2DM, and some evidence demonstrated that a low-carbohydrate diet is sustainable and can lead to diabetes reversal and deprescription [9].

Diabetes-specific nutrition formulas (DSNFs) that offer less total carbohydrate as well as a variation in the type of carbohydrate become essential nutritional support to help minimize the glycemic response, improve outcomes in acute and critical illness, and lower mortality [2,6,10,11]. DSNFs typically comprise 40% carbohydrate, 20% protein, and 40% fat [12]. They are used in diabetes management, as dietary supplements, or occasional meal replacements [12,13]. While several DSNFs are designed for tube feeding and long-term care, the options for ambulatory patients are limited, potentially affecting long-term acceptance and utilization. Moreover, choosing EN formulas should consider patient demographics, clinical conditions, cultural preferences, caloric requirement, and the willingness and ability to make behavioral changes [4].

Although specific protein intake recommendations for diabetic patients are lacking, higher protein diets offer potential benefits. Most T2DM individuals are overweight or obese, and high-protein diets increase satiety and extend post-meal fullness [14,15]. High-protein diets were reported to help lower postprandial plasma glucose in diabetic [16] and in obese subjects with insulin resistance [17]. Soybeans, rich in protein (40%), fat (20%), and dietary fiber (9%), were reported to increase postprandial insulin and glucagon-like peptide-1 (GLP-1), contributing to increased satiety after protein ingestion [18]. In the meta-analysis, several experimental and observational studies suggested that soy protein supplementation could be beneficial for glucose, insulin, the homeostasis model of assessment for insulin resistance index (HOMA-IR), diastolic blood pressure (DBP), low-density lipoprotein cholesterol (LDL-C), total cholesterol (TC), and *C*-reactive protein (CRP) [19]. Additionally, manufacturers of diabetes products suggest that the higher fat and soluble fiber contents slow gastric emptying, thereby preventing fluctuations in blood glucose [5,20].

This study aims to evaluate the impact of two newly developed formulas compared to a commercially available DSNF on glucose–insulin homeostasis, serum GLP-1, serum free fatty acids and serum triglycerides (TG) in obese T2DM patients. Glucerna is a commonly used DSNF from Abbott Nutrition Inc., USA, while the latter two formulas have been recently developed, which contain high protein levels, unsaturated fatty acids, and medium-chain triglycerides (MCTs).

## 2. Materials and Methods

### 2.1. Development of Modified Nutrition-Dense Smoothie Diets

The smoothie drinks were developed using indigenous Thai raw materials rich in macronutrients combined with the essential micronutrients, based on the principles of balanced energy at 1 kcal/mL. We formulated two diabetes-specific smoothies: white sesame soymilk smoothie (SM) and white sesame soymilk smoothie with modified carbohydrate content (SMMC). These recipes had higher protein and fat levels and low glycemic index carbohydrates with the key difference being the substitution of some carbohydrates with eggs, MCTs, and polyunsaturated fatty acids (PUFAs) in the SMMC (Table 1). Protein sources included soybeans, mung beans, eggs, and pea protein. The major fat component included MCTs extracted from coconut oil, monounsaturated fatty acids (MUFAs) from rice bran oil, and PUFAs from soybean oil. The carbohydrates comprised low-dextrose-equivalent maltodextrin, potato, apple juice, and refined sugar.

These smoothie products were produced in the Food Innovation Service Plant (FISP) at Thailand Institute of Scientific and Technological Research (TISTR), Pathum Thani, Thailand. The soybeans and mung beans were soaked in water at a ratio of 1:6 and 1:4 (beans to water, *w*/*w*) for 12 h at 4 °C, respectively, prior to milk extraction. All ingredients were blended together using a high-shear mixer, colloid mill, and homogenizer until a uniform consistency was achieved. The smoothies were sterilized at 116 °C for 62 min in the 180 g retort pouch. For safety purposes, microorganisms (*Salmonella* spp., *Escherichia coli*, *Staphylococcus aureus*, *Bacillus cereus*, *Clostridium* spp., and total coliforms) and other toxic substances (tin, lead, mercury, arsenic, aflatoxin) were tested, and the results showed values within the normal range according to the guidelines of the Thai Food and Drug Administration. In this study, the smoothies were obtained in one batch to maintain homogeneity.

The composition of energy, carbohydrate, protein, fat, and micronutrients was determined by the Asia Medical and Agricultural Laboratory and Research Center, Bangkok, Thailand, according to the AOAC standard protocol [21]. Both the SM and SMMC were normocaloric (1 kcal/mL), hypoglycemic (28–39%), and hyperproteic (24–28%) (Table 2). The SM (300 kcal) provided 18.1 g of protein, 12.3 g of fat, and 28.9 g of carbohydrates, while the SMMC (300 kcal) provided 21.3 g of protein, 14.8 g of fat, and 20.7 g of carbohydrates. Glucerna^®^, a diabetic commercial formula (Abbott Nutrition Inc., Columbus, OH, USA), was used as a control. Glucerna^®^ was prepared from the vanilla powdered formula with a standard 1 kcal/mL recipe according to the manufacturer’s instructions, and it contained 38% carbohydrate, 18% protein, and 33% fat. The smoothie drinks were classified as a low-acid food (pH 6.2–6.5). The viscosities of the SM and SMMC were 194 and 155 centipoise (cP), respectively, which met the International Dysphagia Diet Standardisation Initiative (IDDSI) criteria for extremely thick drinks or pureed foods (Supplementary Table S1). All two nutrition-dense smoothie diets have a nectar-like texture (51–350 cP). Glucerna^®^ (9 cP) is classified as a thin liquid (1–50 cP) [22].

### 2.2. Study Design and Participants

A double-blinded, randomized, crossover clinical study was designed to measure the primary outcome of glycemic responses and the secondary outcomes of metabolic hormones following a bolus of an isocaloric amount of three smoothie drinks. This study recruited 42 adults with T2DM and was conducted at Siriraj Medical Research Center, Siriraj Hospital, Bangkok, Thailand. Eligible participants had T2DM for at least 3 months, were aged 18–65 years, had a body mass index (BMI) ≥ 23 kg/m^2^, had glycated hemoglobin (HbA1c) 6.5–8.5%, and had stable anti-hyperglycemic or lipid-lowering medications for 3 months. Participants were excluded if they met any of the following criteria: treated with insulin or GLP-1 analogues or dipeptidyl peptidase-4 inhibitors (DPP-4 inhibitors), had gastrointestinal disease (such as gastroparesis, malabsorption syndromes), had a history of gastric bypass surgery or tube feeding, were under treatment for endocrinology and kidney disorders, or were pregnant or lactating. The sample size calculation was performed using G*Power software version 3.1, Germany. Relying on the literature effect size of 0.2 in glucose response as a primary outcome [6], 32 patients were required to achieve 80% power at a 5% level of significance. Assuming a dropout rate of 10%, a total sample size of 36 would be required. Written informed consent was obtained from each participant, and this study was approved by the Siriraj Institutional Review Board (COA no. Si 185/2020) and fulfilled all requirements for human research, including the Declaration of Helsinki and Good Clinical Practice. This study’s protocol was registered on clinicaltrials.gov (NCT04577274).

### 2.3. Research Clinic Visits

**Figure 1:**
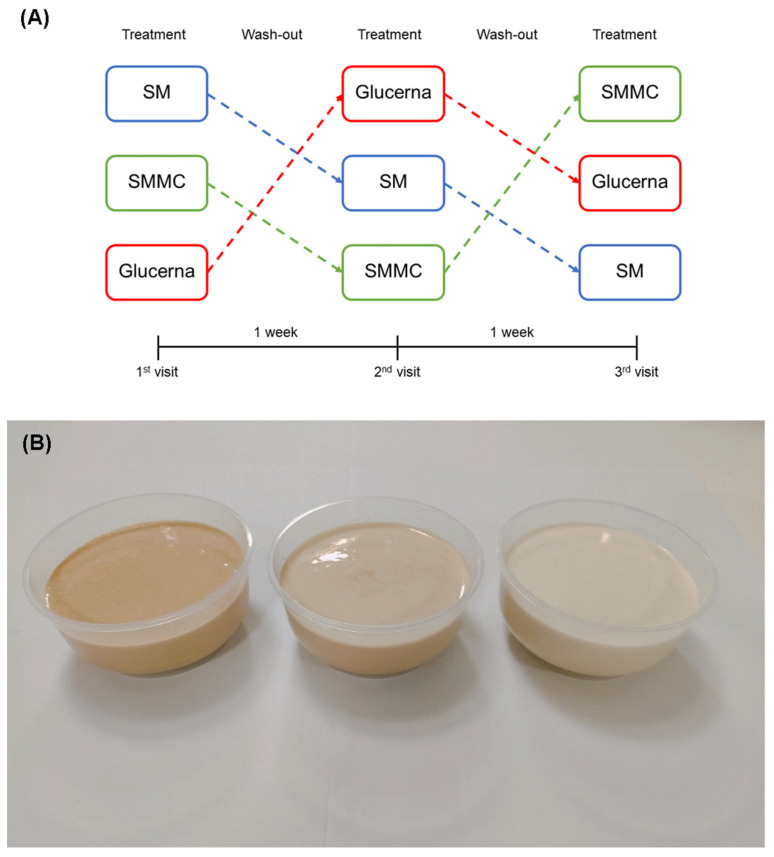
Study design. (**A**) After screening for eligibility, participants were randomized to consume one diet per visit on-site with a one-week washout during the study. (**B**) From left to right, the three types of diet include regular smoothie (SM), smoothie with modified carbohydrate content (SMMC), and Glucerna^®^ (control).

The participants underwent the study procedures on a screening visit and three subsequent visits separated by a 5–7-day washout period between visits (Figure 1A). The visits were conducted in the morning after 8 h of overnight fasting. The sequence of the three diets (SM, SMMC, and Glucerna) was randomly assigned and blind-labeled with random three-digit codes. The study visits were completed within a three-week window starting from the day of the first visit to minimize any variability in dietary and physical activity patterns. The participants were asked to maintain their regular diets and physical activity during the study period. A 24 h food record was collected and examined for energy and macronutrient intake at each visit to confirm the consistency of dietary patterns. These intakes were calculated by using the food composition database in INMUCAL Nutrients software version 4.0 (developed by the Institute of Nutrition, Mahidol University, Nakhon Pathom, Thailand), which is based on Thai food composition and recipes. The total physical activity was obtained with the Thai physical activity questionnaire (ThaiPAQ; MET-min week^−1^) at each visit [23]. The participants were instructed to withhold their anti-hyperglycemic and lipid-lowering medications on the morning of the visit. One of three diets was served as a breakfast at each visit. All diets were equal in caloric content (300 kcal/diet) and served warm at 60 °C (Figure 1B). The participants consumed a bolus of the diets within 5 min and ingested approximately 100 mL of water after consuming the diets.

### 2.4. Anthropometric Assessment

Body weight, excluding footwear, was measured with precision to the nearest 0.1 kg (Tanita HD-395, Tanita Corporation, Tokyo, Japan). Standing height, accurate to the nearest 0.5 cm, was determined with a wall-mounted wooden ruler (Institute of Nutrition, Mahidol University, Nakhon Pathom, Thailand). BMI was calculated as weight divided by the square of height (in kg/m^2^). Waist circumference was measured to the nearest 1.0 cm using a standard measuring tape at a point immediately above the iliac crest on the mid-axillary line. Blood pressure was measured using a blood pressure monitor (HEM-7322, Omron Healthcare Co., Ltd., Kyoto, Japan) at the beginning of the visit for safety. If the blood pressure was >130/80 mmHg, the participants were asked to remain seated and relaxed for 5 min before remeasurement.

### 2.5. Laboratory Assessment

At each test visit, a registered nurse drew a blood sample of approximately 15 mL at baseline, 30, 60, 90, 120, 180, and 240 min from the start of the diet. Blood glucose and HbA1c were measured using the hexokinase method and turbidimetric inhibition immunoassay (TINIA), respectively. Serum insulin levels were analyzed using the sandwich immunoassay with electrochemiluminescence (ECLIA), while lipid profiles were examined using the enzymatic method. Blood samples were immediately centrifuged (1000× *g*, 10 min, 4 °C) to obtain plasma, whereas serum was collected after 30 min of clotting. The specimens were frozen at −80 °C for further analysis. *C*-peptide, leptin, GLP-1, and glucagon were assessed using a bead-based fluorescence assay, the MILLIPLEX^®^ MAP Human Diabetes Magnetic Bead Panel (HDIAB-34K-PMX5; Merck EMD Millipore, Billerica, MA, USA), run on the MAGPIX^®^ instrument. Serum TGs were measured with an enzymatic procedure using GPO-HMMPS, glycerol blanking method (Fujifilm Wako Pure Chemical Corporation, Osaka, Japan). Non-esterified fatty acids (NEFAs) were investigated using the enzymatic colorimetric method (NEFA C, ACS-ACOD method, Fujifilm Wako Pure Chemical Corporation, Osaka, Japan). All samples from the same individual were measured in the same assay run, and quality controls provided by the manufacturer were within allowed limits. After collection of the last sample, the participants were given a meal to eat and were instructed to resume their regular medications.

### 2.6. Statistical Analysis

The sample size was calculated based on the mean difference in the glucose area under the curve (AUC) between the groups from the previous study of Mottalib et al. with the power and alpha level set at 80% and 0.05, respectively [6]. Assuming a dropout rate of 10%, a sample size of 36 participants was considered adequate. The statistical analysis was conducted using SPSS software for Windows (version 19.0, SPSS, Inc., Chicago, IL, USA) and GraphPad Prism 9 (GraphPad Software, San Diego, CA, USA). The normal distribution of the values was checked with the Kolmogorov–Smirnov test. Continuous variables were presented as means and standard error means, while categorical data were presented as numbers and percentages. The AUC was calculated with the trapezoidal formula [24]. The mean differences of measured variables were analyzed using repeated measures analysis of variance (ANOVA) and Latin Square Design (LSD) tests with treatment and time as the fixed effects and subject as the random effect, adjusted for two covariates, including sex and age.

## 3. Results

**Figure 2:**
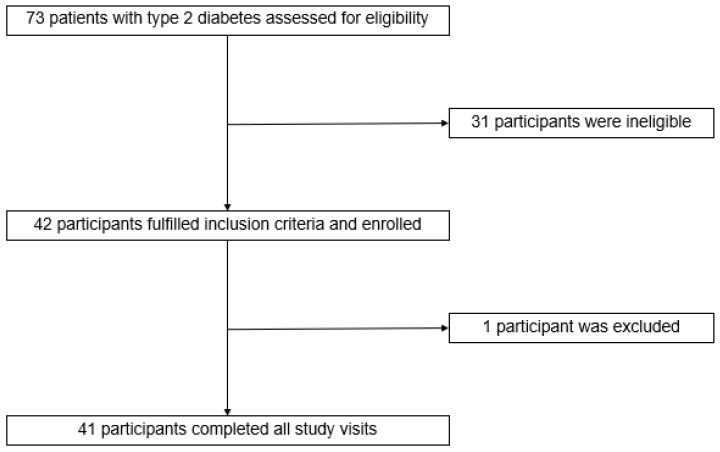
Participant flow diagram.

Out of 73 patients with T2DM screened, 42 participants were enrolled of whom 41 completed all study visits (Figure 2). One participant dropped out due to marked hyperglycemia before entering the second visit. The data from one dropout were excluded from our analysis. The baseline characteristics of the participants are shown in Table 3. The participants had a mean age of 50.0 ± 1.39 years, and 58.5% were female. The average time since diabetes diagnosis among the patients was 6.2 ± 0.95 years, and 87.8% of the participants were on stable diabetic medication, i.e., metformin, glipizide, and pioglitazone, for over three months. Additionally, 63.4% and 41.5% of diabetic subjects had hypertension and dyslipidemia, respectively. The mean BMI of the participants was 30.0 ± 0.80 kg/m^2^. The baseline measurements for HbA1c, fasting blood glucose, and insulin were 7.2 ± 0.12%, 139.2 ± 4.61 mg/dL, and 18.4 ± 3.13 µU/mL, respectively. Daily energy intake, macronutrient consumption, and physical activity in the week before each trial showed no significant differences (Supplementary Table S2).

**Figure 3:**
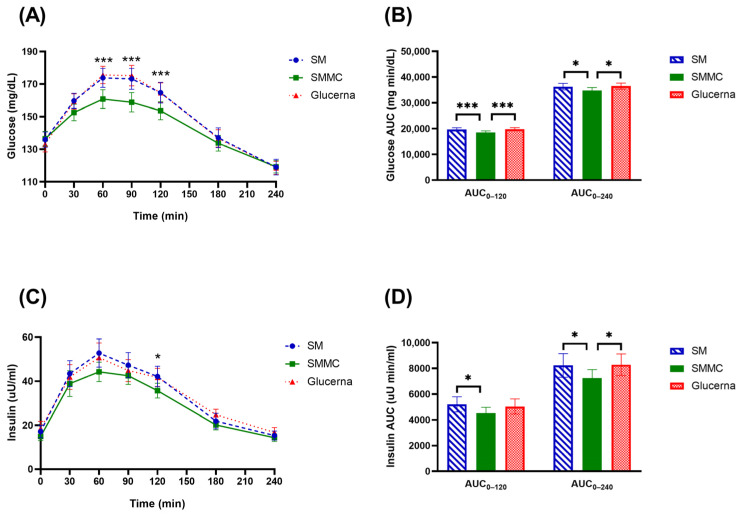
Changes in (**A**) glucose concentrations, (**B**) glucose AUC, (**C**) insulin concentrations, and (**D**) insulin AUC after consumption of the modified nutrition-dense smoothie diets compared to the diabetes commercial formula. Data were analyzed using analysis of variance (ANOVA) and Latin Square Design (LSD) tests with treatment and time as fixed effects and subject as the random effect, adjusted for two covariates, including sex and age. * *p* ≤ 0.05 and *** *p* ≤ 0.001. Abbreviations: AUC, area under the curve; μU/mL, microunit per milliliter; μU min/mL, microunit minute per milliliter; mg/dL, milligram per deciliter; mg min/dL, milligram minute per deciliter; SM, regular smoothie drink; SMMC, smoothie with modified carbohydrate content.

The change in glucose and insulin concentrations from baseline over 240 min for each formula is depicted in Figure 3A (Supplementary Table S3). The baseline mean plasma glucose and insulin concentrations were comparable for all three formulas (*p* = 0.500). Compared to the baseline (0 min), there was an increase in blood glucose concentration in response to a dietary challenge with Glucerna and the SM formula, which lasted until 120–180 min. On the contrary, only moderate increases in blood glucose concentration from baseline occurred at 60 min with the SMMC formula with no further increase in blood glucose across time. Between the formulas, the increase in blood glucose was significantly lower when the participants were given the SMMC formula compared to the SM and Glucerna formulas across three time points from 60 to 120 min (*p* ≤ 0.001). The peak glucose concentration of Glucerna was 175.54 ± 5.38 mg/dL at 60 min, while the peak glucose concentrations of SM and SMMC consumption were 173.76 ± 5.79 and 160.76 ± 5.77 mg/dL, respectively. Blood glucose returned to the baseline level at 180 min with all three formulas. Glucose positive AUC_0–120_ and positive AUC_0–240_ for the SMMC were significantly lower than those for the SM and Glucerna (*p* < 0.05) (Figure 3B).

The change in insulin concentration from baseline over 240 min by formula is shown in Figure 3C. The mean insulin concentrations were not significantly different at baseline between the formulas, 17.20 ± 2.82, 14.97 ± 1.92, and 18.64 ± 3.21 µU/mL, for the SM, the SMMC, and Glucerna, respectively (*p* = 0.055). There was a trend towards lower mean endogenous insulin production in response to the SMMC group over time (30–240 min) when compared to the SM and Glucerna groups, while the insulin level at 120 min of the SMMC was significantly lower when compared to that of the SM and Glucerna groups (*p* < 0.05). The maximums of the average insulin were 44.29 ± 4.44, 52.82 ± 6.41, and 50.79 ± 6.65 µU/mL for the SMMC, the SM, and Glucerna formulas, respectively. The serum insulin gradually decreased postprandially to the baseline at 180 min after each formula. Insulin positive AUC_0–240_ for the SMMC was significantly lower than that for the SM and Glucerna (*p* < 0.05) (Figure 3D).

**Figure 4:**
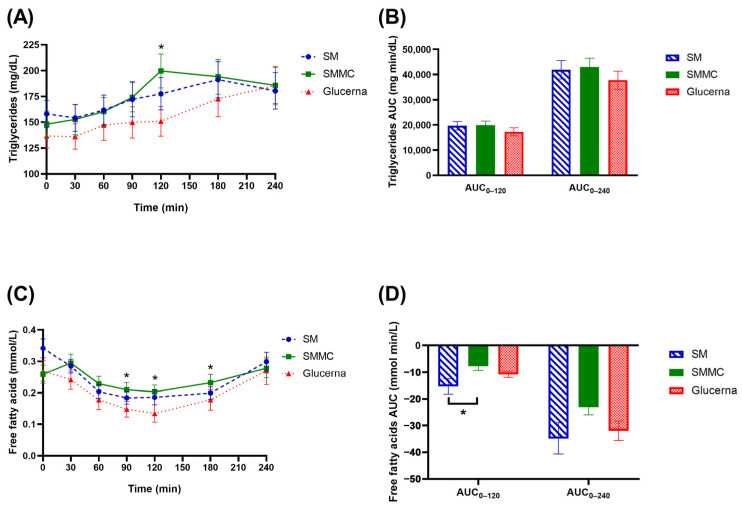
Changes in (**A**) triglyceride concentrations, (**B**) triglyceride AUC, (**C**) free fatty acid concentrations, and (**D**) free fatty acid AUC after consumption of the modified nutrition-dense smoothie diets compared to the diabetes commercial formula. Data were analyzed using analysis of variance (ANOVA) and Latin Square Design (LSD) tests with treatment and time as fixed effects and subject as the random effect, adjusted for two covariates, including sex and age. * *p* ≤ 0.05. Abbreviations: AUC, area under the curve; mg/dL, milligram per deciliter; mg min/dL, milligram minute per deciliter; mmol/L, millimole per liter; mmol min/L, millimole minute per liter; SM, regular smoothie drink; SMMC, smoothie with modified carbohydrate content.

Figure 4A displays the alteration in TG concentration from baseline over a 240 min period by formula. Fasting TG on the SM versus SMMC versus Glucerna exhibited no significant difference (158.11 ± 13.25, 148.11 ± 12.73, and 136.80 ± 11.83 mg/dL, respectively). The peak serum TG concentration was attained 120 min after SMMC consumption, while the peak TG concentrations were reached 180 and 240 min after consuming the SM and Glucerna formulas, respectively. The ingestion of the SMMC amplified the increase in serum TG compared with the SM and Glucerna diets, which tended to have a higher total AUC_0–120_ and AUC_0–240_ (Figure 4B). Fasting NEFA concentrations exhibited no significant difference among the three formulas (Figure 4C). Significant differences were observed in the NEFA responses following the test meals with the SMMC meal showing a different pattern of response compared with the Glucerna meals. Following the test meals, NEFA concentrations were initially suppressed, reaching a nadir at approximately 120 min. During the diets, the suppression of NEFA was more augmented on the SM, resulting in a less total AUC_0–120_ of NEFA compared with the SMMC diet (*p* < 0.05; Figure 4D) but not the Glucerna diet. Nadir NEFA concentration with the SMMC diet was 0.20 ± 0.02 mmol/L at 120 min compared with 0.13 ± 0.03 mmol/L with the Glucerna diet (*p* = 0.012). At the end of the postprandial period at 240 min, no different NEFA concentrations were observed among the three formulas.

**Figure 5:**
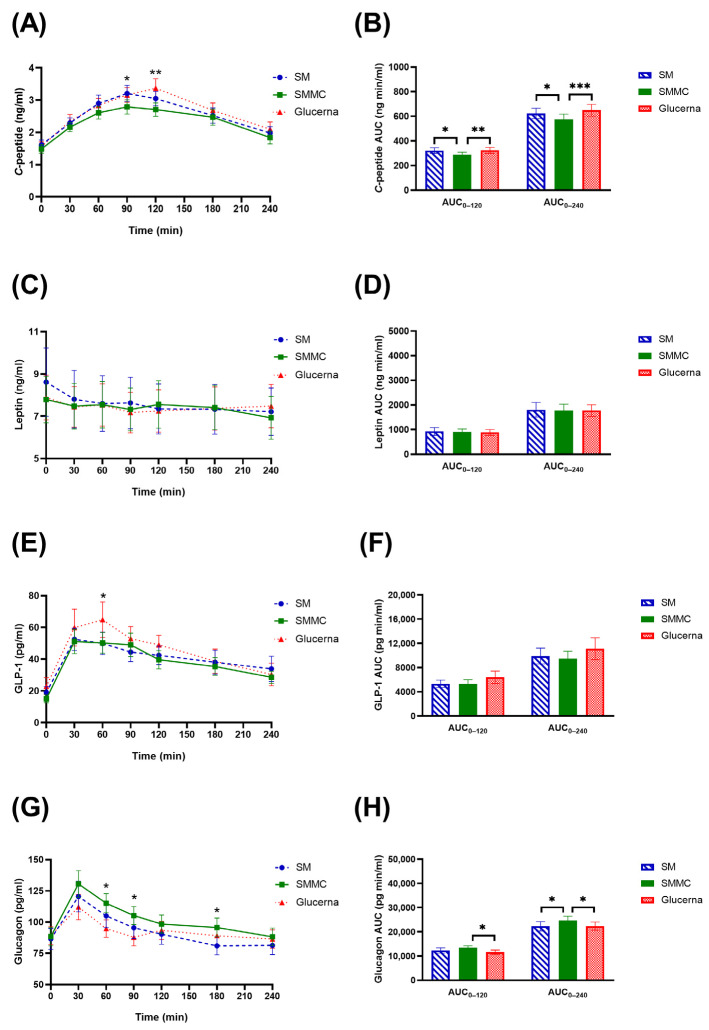
Changes in (**A**) *C*-peptide concentrations, (**B**) *C*-peptide AUC, (**C**) leptin concentrations, (**D**) leptin AUC, (**E**) GLP-1 concentrations, (**F**) GLP-1 AUC, (**G**) glucagon concentrations, and (**H**) glucagon AUC after consumption of the modified nutrition-dense smoothie diets compared to the diabetes commercial formula. Data were analyzed using analysis of variance (ANOVA) and Latin Square Design (LSD) tests with treatment and time as fixed effects and subject as the random effect, adjusted for two covariates, including sex and age. * *p* ≤ 0.05, ** *p* ≤ 0.01, *** *p* ≤ 0.001. Abbreviations: AUC, area under the curve; ng/mL, nanogram per milliliter; ng min/mL, nanogram minute per milliliter; pg/mL, picogram per milliliter; pg min/mL, picogram minute per milliliter; SM, regular smoothie drink; SMMC, smoothie with modified carbohydrate content.

Fasting *C*-peptide on the SM versus SMMC versus Glucerna exhibited no significant difference (Figure 5A, Supplementary Table S4). The peak of *C*-peptide was attained 90 min after the SMMC and SM and 120 min after Glucerna consumption, respectively. *C*-peptide total AUC_0–120_ and AUC_0–240_ for the SMMC were significantly lower than those for Glucerna (*p* < 0.01) (Figure 5B). There was no significant difference in both leptin concentration at all timepoints and leptin AUC_0–240_ between each diet (Figure 5C,D). Plasma GLP-1 concentrations increased almost three-fold within 1 h after each meal. The GLP-1 peak secretion time was 60 min after Glucerna consumption, while the GLP-1 peak secretion time was 30 min after SM and SMMC consumption, respectively (Figure 5E). Postprandial GLP-1 was significantly different at 60 min after SMMC consumption in comparison to that for Glucerna (*p* = 0.019). The total AUC of GLP-1 exhibited a statistically insignificant difference among the groups (Figure 5F). There was a trend towards higher mean endogenous glucagon production in response to the SMMC group over time (30–240 min) when compared to the SM and Glucerna groups (Figure 5G). Glucagon total AUC_0–120_ and AUC_0–240_ for the SMMC were significantly higher than those for Glucerna (*p* < 0.05) (Figure 5H).

## 4. Discussion

This study aimed to compare the glycemic and insulinemic responses of three specialized nutritional formulas in patients with T2DM. The glucose responses to the formulas showed a significant increase in blood glucose concentration in response to a dietary challenge with Glucerna and the SM formula, which lasted until 120–180 min. On the contrary, only moderate increases in blood glucose concentration from baseline occurred at 60 min with the SMMC formula. The endogenous insulin responses showed a trend towards lower mean insulin production in response to the SMMC group over time compared to the SM and Glucerna groups. The maximum average insulin was 44.3 µU/mL for the SMMC formula, significantly lower than that for the SM and Glucerna formulas. The change in TG concentration from baseline showed that the increase in serum TG was enhanced after ingestion of the SMMC compared with the SM and Glucerna diets, leading to a tendency toward higher total AUC_0–120_ and AUC_0–240_. However, the suppression of NEFA was more augmented on the SM, resulting in a lower total AUC_0–240_ of NEFA compared with the SMMC diet but not the Glucerna diet.

This study demonstrated that the SMMC formula yields a favorably reduced postprandial glycemic and insulinemic response compared to the SM and Glucerna formulas. The lower peak glucose and insulin concentrations and lower glucose and insulin positive AUC_0–120_ and positive AUC_0–240_ observed in the SMMC group may be due to the presence of a lower amount of carbohydrate or a higher amount of low glycemic index carbohydrate, protein, and fat, which made them absorb more slowly in the SMMC formula. Fiber and high proportions of protein and fat from beans, eggs, and isolated soy protein may decrease the gastric emptying rate and decrease the rate and degree of glucose absorption. Meal enrichment with soluble dietary fiber and protein has been shown to decrease postprandial blood glucose and fasting blood glucose and increase insulin response, which may be caused by increased food viscosity from the additional natural protein [25,26]. Given these findings, it is reasonable to postulate that the improved insulin sensitivity observed with the SMMC formula may have positive implications for long-term diabetes management. Enhanced insulin sensitivity is associated with improved glucose control and may consequently contribute to a decreased risk of diabetes-related complications. While further research is warranted to validate these postulations, our study lays the groundwork for considering the potential long-term benefits of dietary interventions like the SMMC formula in individuals with diabetes.

In addition, GLP-1, the incretin hormone secreted from the gastrointestinal tract to stimulate insulin secretion from the pancreas, tends to decrease during the first 30–60 min after SM and SMMC consumption. Glucagon, a counterregulatory hormone for insulin, tends to increase after SM and SMMC consumption compared to Glucerna consumption. The administration of a meal typically leads to an increase in both insulin and glucagon levels, and this response is the regulatory mechanism to maintain blood glucose homeostasis. In addition, glucagon can activate specific G-protein-coupled receptors on pancreatic beta cells, leading to stimulation of adenylate cyclase and, subsequently, activation of insulin secretion [27,28]. A recent study suggested that the rise in insulin release following an elevation in glucagon levels could be attributable to both heightened glucagon-induced glucose production and a direct impact on the secretion of insulin from beta cells [29]. It is noteworthy that the overall response of glucagon and insulin in the Glucerna and SM groups was comparable. Insulin’s primary role is to facilitate the uptake of nutrients, particularly glucose, by cells. After a meal, an increase in blood glucose levels triggers the release of insulin to promote the utilization and storage of the incoming nutrients [30]. The SMMC may attenuate the insulin requirement for glucose uptake compared to the SM and Glucerna because of a lower amount of carbohydrate. Despite the increase in insulin after a meal, the body also needs to counterbalance the potential drop in blood glucose levels caused by increased cellular uptake. Glucagon is released to prevent hypoglycemia by promoting the release of glucose from storage forms like glycogen [31]. The higher glucagon response after SMMC consumption may have been a homeostatic response to the lowest glucose response. Another reason for the highest glucagon response after SMMC consumption may be due to the higher amount of protein, which has also been noted by other studies [32,33]. However, postprandial leptin did not show a significant difference after SMMC consumption in comparison to Glucerna in accordance with previous research, which reported that a carbohydrate-reduced, high-protein diet decreases postprandial glucose excursions in T2DM patients [34].

These findings are consistent with those reported by Jackson et al. who indicate that the nature of the fatty acids ingested during meals influences the initial rise of NEFA and TG levels [35]. Compared with a meal containing short-chain fatty acids (SCFAs), a meal containing unsaturated fatty acids showed a lower net incremental AUC for NEFA response. Similarly, diets containing the SMMC, a mixture of fatty acids, led to a lower net incremental glucose and insulin response. This reflects the relative contribution of NEFA from adipose tissue lipolysis, the release of NEFA from circulating TG-rich lipoproteins, and uptake of NEFA into the peripheral tissues and liver. Notably, the postprandial concentrations of both TG and NEFA were similar at 240 min after consuming all three meals. TG may originate from dietary sources (exogenous pathway) or be synthesized by the liver (endogenous pathway). Medium-chain fatty acids (MCFAs) from the MCTs, one of the components in the SM and SMMC, are absorbed directly through the villi of the intestinal mucosa and transported to the liver via portal circulation without being incorporated into chylomicrons. In contrast, long-chain fatty acids (LCFAs) from vegetable oils in smoothies and Glucerna follow a more complex metabolic pathway that encompasses the synthesis of chylomicrons in the intestinal villi [36]. Chylomicrons are present in only plasma following a high-fat diet consumption to transport the TG obtained from food through the lymphatic system into the bloodstream to adipose tissues and muscles [37]. Hydrolysis of MCT to its component fatty acids is faster and more efficacious than that of LCFAs [38]. It is suggested that the fat content in the SMMC is converted to TG upon consumption, leading to an increase in serum TG. The SMMC has a higher ratio of MCFAs to LCFAs than the SM and Glucerna, resulting in the rapid modification of TG. TG is rapidly absorbed into the bloodstream and taken up by the liver, leading to a decrease in TG levels after 120 min of SMMC consumption. These TG levels return to similar levels as compared with Glucerna at 240 min. Normally, plasma TG levels increase after a fat-containing meal and return to baseline 6–12 h later [39].

During the fasting state, free fatty acids are released into the bloodstream by fat breakdown in subcutaneous adipose tissue. Obese people tend to have more visceral adipose tissue, resulting in more free fatty acids circulating in the bloodstream as well [40]. In the fed state, insulin inhibits lipolysis and increases re-esterification of free fatty acids. The NEFA concentration at 90, 120, and 180 min after Glucerna consumption was significantly lower than that after SMMC consumption (*p* < 0.05 each). The nadir NEFA AUC_0–120_ for the SMMC was significantly higher than that of the SM (*p* < 0.05), while there was no significant difference between the SMMC and Glucerna during the first 2 h. Mottalib et al. noted that the high fat content in the diet may affect the inhibition of that mechanism’s expression [6]. The results of both TG and free fatty acids in this study are consistent with the study by Samkani et al., which compared TG and free fatty acids between the carbohydrate-reduced, high-protein (CRHP) diet and the conventional diabetes (CD) diet [41]. They found that CRHP consumption provided higher TG and free fatty acids than CD consumption at breakfast. Interestingly, they found that CRHP consumption provided lower TG and free fatty acids than CD consumption after lunch. Therefore, further study on the effectiveness of long-term smoothie consumption may provide more obvious changes. Monitoring of the reduction in kidney and cardiovascular complications needs to be considered.

The postprandial GLP-1 and leptin responses to meal stimulation depend partially on circulating glucose absorption, and GLP-1 promotes insulin release in humans [42,43]. GLP-1 may also decrease food intake and increase energy expenditure [44]. However, we did not observe a significant difference for these markers. This is probably due to the short-term effects of meal ingestion. Gonzalez et al. showed that changes in plasma leptin and glucagon concentrations were observed over 24 h among interventions [45], since leptin is thought to be mostly regulated by chronic changes in energy balance and plays a role in suppressing appetite. Therefore, long-term studies are needed to verify the effect of these smoothies on this peptide hormone.

This study has some limitations. This study was an acute intervention with a relatively small number of participants at only one medical facility. While our study primarily focuses on short-term results, we recognize the need for caution in generalizing these findings to long-term scenarios. We acknowledge that extrapolating these results to long-term outcomes may require additional research with extended follow-up periods. Additionally, the population may not allow for the generalization of the results to younger populations. We deliberately chose this age group based on specific criteria related to the targeted health condition and potential influences on the study outcomes. Type 2 diabetes most often develops in people over age 45 years [46]. Future studies involving a more diverse age distribution will be considered to broaden the applicability of our results.

As there was no significant difference in the metabolic factors between the SM and Glucerna, it showed that the SM was sufficiently comparable to the standard diabetic formula. Previous studies compared the normal diet and the standard diabetic diet and reported that the standard diabetic diet often provided better glycemic results [47,48]. The SMMC was noticeably better than the SM and Glucerna because it offered a better glucose and insulin response. Therefore, the SMMC may be a good candidate as a promising functional food for management and supplementation for T2DM and obese people in the future.

## 5. Conclusions

The short-term postprandial metabolic changes observed in this crossover study indicated that the SMMC was superior to the conventional diabetic enteral drink in reducing the postprandial glucose response in overweight and obese patients with T2DM, while the SM had no significant differences on metabolic changes when compared to Glucerna. This study’s findings may have implications for the selection of specialized nutritional formulas for patients with T2DM, considering their glycemic, insulinemic, and lipidemic responses. Further research is needed to confirm these findings and investigate the long-term effects of different specialized nutritional formulas on glycemic and lipidemic control in patients with T2DM.

## Figures and Tables

**Table 1:** Ingredients of the developed formulas.

Ingredients/100 g	SM	SMMC
Macronutrients		
Soybean milk (g)	35	35
Mungbean milk (g)	23	23
Egg (g)	2	10
Soy isolated protein: Pea protein (ratio)	2:2.5	2:2.5
Apple juice (g)	10	10
Potato (g)	5	2
Maltodextrin (g)	3	2
Sugar (g)	3	2
Sesame (g)	2	2
SFA:MUFA:PUFA (ratio)	2:1:0	3:2:1
Vitamin Premix		
Vitamin A (µg retinol)	154	154
Thiamin (mg)	0.3	0.3
Riboflavin (mg)	0.4	0.4
Niacin (mg)	3.4	3.4
Pantothenic acid (mg)	1	1
Vitamin B6 (mg)	0.4	0.4
Vitamin B12 (µg)	0.4	0.4
Vitamin C (mg)	19	19
Cholecalciferol (µg)	1	1
α-Tocopherol (mg)	1.9	1.9
Phytonadione (µg)	18	18
Biotin (µg)	25	25
Folic acid (µg)	44	44

Abbreviations: g, gram; mg, milligram; µg, microgram; MUFA, monounsaturated fatty acid; PUFA, polyunsaturated fatty acid; SFA, saturated fatty acid; SM, regular smoothie drink; SMMC, smoothie with modified carbohydrate content.

**Table 2:** Composition of modified nutrition-dense smoothie diets and diabetes commercial formula.

	SM ^1^ (285 g)	SMMC ^1^ (290 g)	Glucerna^®^ (Control) ^2^ (300 g)
Energy (kcal)	300	300	300
Carbohydrate (g, %)	28.9, 39	20.7, 28	28.5, 38
Protein (g, %)	18.1, 24	21.3, 28	13.5, 18
Fat (g, %)	12.3, 37	14.8, 44	11.0, 33
Saturated fat (g)	5.5	6.1	N/A ^3^
Cholesterol (mg)	16.4	46.2	N/A ^3^
Sugar (g)	12.1	9.3	N/A ^3^
Sodium (mg)	151.4	186.4	280.7
Vitamin A (µg retinol)	404.4	400.2	735.3
Vitamin B1 (mg)	0.9	0.8	0.5
Vitamin B2 (mg)	1.5	1.7	0.6
Calcium (mg)	68.6	72.8	223.8
Iron (mg)	2.3	2.3	1.8

^1^ Analysis from Asia Medical and Agricultural Laboratory and Research Center Co., Ltd.; ^2^ Data from Abbott Nutrition (prepared from Glucerna^®^ powder 69 g in water 231 g); ^3^ N/A means not available; Abbreviations: g, gram; kcal, kilocalorie; mg, milligram; µg, microgram; SM, regular smoothie drink; SMMC, smoothie with modified carbohydrate content.

**Table 3:** Baseline characteristics of participants who attended the study.

Measures (Mean ± Standard Error Mean)	n = 41
Age (years)	50.0 ± 1.39
Male, n (%)	17 (41.5)
Female, n (%)	24 (58.5)
Underlying diseases ^1^	
Diabetes Mellitus, n (%)	41 (100.0)
Diabetes duration (years)	6.2 ± 0.95
Hypertension	26 (63.4)
Dyslipidemia	17 (41.5)
Body weight (kg)	78.3 ± 2.30
Height (cm)	161.5 ± 1.28
BMI (kg/m^2^)	30.0 ± 0.80
Waist circumference (cm)	100.2 ± 1.71
Systolic blood pressure (mmHg)	132.9 ± 2.16
Diastolic blood pressure (mmHg)	85.4 ± 1.45
HbA1c (%)	7.2 ± 0.12
Fasting blood glucose (mg/dL)	139.2 ± 4.61
Fasting insulin (μU/mL)	18.4 ± 3.13
Triglyceride (mg/dL)	173.7 ± 17.89
Cholesterol (mg/dL)	176.8 ± 7.33
HDL Cholesterol (mg/dL)	50.4 ± 1.75
LDL Cholesterol (mg/dL)	114.0 ± 6.64

^1^ Participants may have more than 1 underlying disease. Abbreviations: BMI, body mass index; cm, centimeter; HbA1c, glycated hemoglobin; kg, kilogram; kg/m^2^, kilogram per square meter; μU/mL, microunit per milliliter; mg/dL, milligram per deciliter; mmHg, millimeters of mercury.

## Data Availability

The data presented in this study are available on request from the corresponding author. The data are not publicly available due to privacy and ethical restrictions.

## Supplementary Materials

The following supporting information can be downloaded at: https://www.mdpi.com/article/10.3390/nu16030395/s1, Table S1: Physical properties of modified nutrition-dense smoothie diets and diabetes commercial formula. Table S2: The comparison of confounding factors that may affect changes in blood glucose and insulin levels. Table S3: Changes in glucose, insulin, triglycerides, and free fatty acid concentrations after consumption of the modified nutrition-dense smoothie diets compared to the diabetes commercial formula. Table S4: Changes in *C*-peptide, leptin, GLP-1, and glucagon concentrations after consumption of the modified nutrition-dense smoothie diets compared to the diabetes commercial formula.
