# Supplementary material for: Diabetes-Specific Complete Smoothie Formulas Improve Postprandial Glycemic Response in Obese Type 2 Diabetic Individuals: A Randomized Crossover Trial
Source: Nutrients. 2024 Jan 30;16(3):395. doi: 10.3390/nu16030395 (PMC10857113; doi:10.3390/nu16030395)
Supplement: Supplementary file 1 [file nutrients-16-00395-s001.zip › nutrients-2845147-supplementary.pdf]

Table S1. Physical properties of modified nutrition-dense smoothie diets and diabetes commercial formula

	**Color**[1]			**pH**[2]	**Viscosity (Centipoise)**[3]
	L*	a*	b*		
**SM**	64.58 ± 0.07	6.88 ± 0.10	26.78 ± 0.27	6.04 ± 0.01	193.99 ± 17.59
**SMMC**	66.44 ± 0.41	6.93 ± 0.02	26.93 ± 0.21	6.17 ± 0.00	154.82 ± 9.73
**Glucerna® (control)**	78.59 ± 0.05	0.36 ± 0.02	13.95 ± 0.11	6.14 ± 0.00	9.00 ± 0.10

[1] Measurement by using Color measurement spectrophotometer (Model Color Quest XE, Hunter lab, USA)

[2] Measurement by using pH meter (Model SevenCompact, Mettler-Toledo, Switzerland)

[3] Measurement by using Coaxial spindle CCT-40, Rheometer (Model RST-CC Touch™, Brookfield Engineering Laboratories, Inc, USA)

Abbreviations: SM, regular smoothie drink; SMMC, smoothie with modified carbohydrate content

Table S2. The comparison of confounding factors that may affect changes in blood glucose and insulin levels[1]

	SM	SMMC	Glucerna	P-value[2]
Energy from food intake in 1 day before attend the visit (kcal)	1,318.0 ± 73.58	1,256.5 ± 68.54	1,326.0 ± 76.99	0.60
Carbohydrate from food intake in 1 day before attend the visit (g)	163.7 ± 11.53	145.7 ± 9.87	154.2 ± 9.95	0.19
Protein from food intake in 1 day before attend the visit (g)	60.8 ± 4.05	58.0 ± 3.18	65.0 ± 7.07	0.50
Fat from food intake in 1 day before attend the visit (g)	46.7 ± 3.82	49.1 ± 3.82	49.9 ± 3.65	0.80
Physical activity in 7 day before attend the visit (MET-min/week)	8,030.6 ± 1,064.40	7,663.1 ± 1,004.03	7,592.3 ± 957.90	0.42

[1] Data are presented as means ± SEM

[2] Obtained from repeated measures analysis of variance (ANOVA)

Abbreviations: g, gram; kcal, kilocalorie; SM, regular smoothie drink; SMMC, smoothie with modified carbohydrate content

Table S3. Changes of glucose, insulin, triglycerides, and free fatty acids concentrations after consumption of the modified nutrition-dense smoothie diets compared to the diabetes commercial formula.

Measures (mean±SEM)	Smoothies	0 min	30 min	60 min	90 min	120 min	180 min	240 min	AUC 0–120 min	AUC 0–240 min
**Glucose (mg/dL)**	**SM**	135.98±4.67	159.68±4.63	173.76±5.79	173.20±6.44	164.73±6.35	137.10±6.05	119.05±4.83	19,709.63±640.84	36,243.29±1,276.04
	**SMMC**	136.49±4.46	152.54±5.05	160.76±5.77	158.83±5.86	153.61±5.48	133.73±4.80	119.24±3.81	18,515.12±635.76	34,724.63±1,166.20
	**Glucerna**	133.15±4.83	159.27±4.69	175.54±5.38	175.20±6.29	164.66±6.11	136.68±5.32	118.59±4.07	19,767.07±620.75	36,465.37±1,198.88
**P-value[1]**		0.500	0.058	**<0.001**	**<0.001**	**0.001**	0.458	0.929	**0.001**	**0.026**
**Insulin (µU/ml)**	**SM**	17.20±2.82	43.46±5.90	52.82±6.41	47.31±5.77	42.18±4.68	21.91±3.51	15.36±2.09	5,198.52±585.66	8,225.58±922.06
	**SMMC**	14.97±1.92	38.91±5.84	44.29±4.44	42.51±3.96	35.72±3.35	20.17±2.23	14.36±1.73	4,531.70±441.97	7,244.49±658.24
	**Glucerna**	18.64±3.21	41.91±5.67	50.79±6.65	44.87±4.99	41.77±4.10	24.81±2.46	16.76±2.25	5,033.07±586.00	8,277.58±846.79
**P-value[1]**		0.055	0.334	0.068	0.342	**0.028**	0.070	0.072	**0.039**	**0.023**
**Triglyceride (mg/dL)**	**SM**	158.11±13.25	154.27±13.00	161.85±14.66	172.23±17.11	177.69±15.64	191.25±17.52	180.47±17.69	19,687.64±1,683.37	41,907.94±3,682.97
	**SMMC**	148.11±12.73	152.62±14.70	160.34±13.75	174.17±14.20	199.75±16.54	194.10±16.90	185.63±17.46	19,831.82±1,636.33	43,038.94±3,512.86
	**Glucerna**	136.80±11.83	136.14±12.19	147.37±14.86	149.87±15.09	150.92±14.26	172.80±17.45	185.55±18.63	17,317.29±1,615.63	37,779.70±3,576.48
**P-value[1]**		0.394	0.450	0.828	0.336	**0.012**	0.525	0.820	0.347	0.363
**Free fatty acid (mmol/L)**	**SM**	0.34±0.03	0.29±0.02	0.20±0.02	0.18±0.02	0.19±0.02	0.20±0.02	0.30±0.03	-15.28±2.89	-34.78±5.83
	**SMMC**	0.26±0.03	0.29±0.03	0.23±0.02	0.21±0.02	0.20±0.02	0.23±0.03	0.28±0.03	-7.72±1.61	-23.08±2.92
	**Glucerna**	0.27±0.03	0.24±0.03	0.18±0.03	0.15±0.02	0.13±0.03	0.18±0.03	0.27±0.04	-10.79±1.11	-31.90±3.61
**P-value[1]**		0.072	0.340	0.141	**0.014**	**0.030**	**0.044**	0.418	**0.049**	0.295

[1] Data were analyzed using Analysis of Variance (ANOVA) and Latin Square Design (LSD) Tests with treatment and time as fixed effects and subject as random effect, adjusted for two covariates, including sex and age. Abbreviations: AUC, area under the curve; SM, regular smoothie drink; SMMC, smoothie with modified carbohydrate content

Table S4. Changes of *C*-peptide, leptin, GLP-1 and glucagon concentrations after consumption of the modified nutrition-dense smoothie diets compared to the diabetes commercial formula.

Measures (mean±SEM)	Smoothies	0 min	30 min	60 min	90 min	120 min	180 min	240 min	AUC 0–120 min	AUC 0–240 min
***C*-peptide**	**SM**	1.61±0.15	2.29±0.16	2.90±0.26	3.21±0.25	3.05±0.22	2.52±0.24	1.98±0.20	321.90±22.35	623.60±42.46
**(ng/ml)**	**SMMC**	1.49±0.13	2.16±0.14	2.61±0.19	2.79±0.22	2.71±0.21	2.47±0.25	1.84±0.20	289.61±20.18	574.22±44.27
	**Glucerna**	1.56±0.14	2.35±0.20	2.84±0.22	3.16±0.23	3.37±0.29	2.68±0.23	2.10±0.22	324.45±23.81	649.36±47.67
**P-value[1]**		0.573	0.145	0.121	**0.020**	**0.008**	0.237	0.072	**0.014**	**0.003**
**Leptin**	**SM**	8.62±1.61	7.81±1.36	7.60±1.31	7.63±1.22	7.35±1.18	7.33±1.17	7.22±1.12	930.42±156.08	1,807.53±292.91
**(ng/ml)**	**SMMC**	7.79±1.10	7.48±1.08	7.55±1.10	7.32±1.01	7.56±1.12	7.41±1.05	6.93±1.02	900.35±128.01	1,779.53±253.30
	**Glucerna**	7.89±1.06	7.44±0.96	7.53±1.00	7.18±0.96	7.25±1.00	7.37±1.02	7.48±1.02	891.50±116.70	1,775.90±235.75
**P-value[1]**		0.651	0.878	0.936	0.839	0.984	0.999	0.726	0.904	0.960
**GLP-1**	**SM**	18.97±3.70	52.40±7.05	49.98±7.00	44.41±6.03	42.44±5.90	38.02±7.50	33.88±7.95	5,324.88±613.35	9,895.83±1,305.68
**(pg/ml)**	**SMMC**	14.97±2.49	51.10±7.58	50.25±6.58	49.06±7.44	39.61±5.73	35.38±5.53	28.56±3.72	5,330.99±666.53	9,498.79±1,185.29
	**Glucerna**	22.87±5.35	59.92±11.59	64.80±11.18	52.86±7.83	49.02±5.95	38.69±7.64	30.37±7.13	6,405.82±1,014.43	11,109.00±1,783.02
**P-value[1]**		0.366	0.327	**0.033**	0.217	0.252	0.634	0.724	0.067	0.149
**Glucagon**	**SM**	86.58±8.42	120.51±12.35	105.00±9.51	95.49±8.66	90.35±8.12	81.03±7.23	81.38±7.33	12,284.06±1,108.60	22,297.59±1,941.82
**(pg/ml)**	**SMMC**	88.44±7.43	130.64±10.72	115.10±7.68	105.28±7.26	98.47±7.24	95.67±7.65	88.26±7.05	13,334.30±908.94	24,676.62±1,706.92
	**Glucerna**	89.23±7.05	112.16±10.18	94.91±7.07	87.84±6.90	93.43±7.25	89.11±7.21	86.46±7.37	11,587.13±892.25	22,330.78±1,684.29
**P-value[1]**		0.782	0.076	**0.024**	**0.015**	0.184	**0.021**	0.191	**0.030**	**0.027**

[1] Data were analyzed using Analysis of Variance (ANOVA) and Latin Square Design (LSD) Tests with treatment and time as fixed effects and subject as random effect, adjusted for two covariates, including sex and age. Abbreviations: AUC, area under the curve; SM, regular smoothie drink; SMMC, smoothie with modified carbohydrate content
